# Home-based transcranial direct current stimulation (tDCS) for bipolar depression: effects on quality of life and functioning—an open-label study

**DOI:** 10.1007/s11136-025-04135-2

**Published:** 2026-01-09

**Authors:** Hakimeh Rezaei, Rachel D. Woodham, Ali-Reza Ghazi-Noori, Elvira Bramon, Michael Bauer, Allan H. Young, Cynthia H. Y. Fu, Philipp Ritter

**Affiliations:** 1 Department of Psychiatry and Psychotherapy, Faculty of Medicine, Technische Universität Dresden, Haus 25, Fetscherstraße 74, 01307 Dresden, Germany; 2 Institute of Psychiatry, Psychology and Neuroscience, King’s College London, London, UK; 3 School of Psychology, University of East London, London, UK; 4 Department of Psychiatry, University College London, London, UK; 5 National Institute for Health Research Biomedical Research Centre at South London and Maudsley NHS Foundation Trust, King’s College London, London, UK; 6 South London and Maudsley NHS Foundation Trust, Bethlem Royal Hospital, Beckenham, UK

**Keywords:** Transcranial direct current stimulation, Bipolar depression, Quality of life, Home‐based treatment

## Abstract

**Purpose:**

Individuals with bipolar disorder often experience reduced quality of life (QoL). Transcranial direct current stimulation (tDCS) is a promising non-invasive treatment for bipolar depression that is portable, safe, and suitable for use at home. We developed a home-based tDCS protocol with real-time remote supervision and examined its effect on QoL in bipolar depression.

**Methods:**

In an open‐label design, 44 participants (31 women) with bipolar depression of at least a moderate severity received 21 sessions of home‐based tDCS (2 mA, 30 min, F3 anode/F4 cathode) over 6 weeks, with a follow-up visit conducted 5 months from baseline. QoL was assessed using the quality of life enjoyment and satisfaction questionnaire (Q-LES-Q) at baseline, week 2, end of treatment, and follow-up session. Baseline and post treatment scores were compared with healthy control participants (28 adults; 17 women).

**Results:**

At baseline and at the end of treatment, bipolar participants showed a significantly lower Q-LES-Q score than healthy controls (*p* < 0.001). Within the bipolar group, there was a significant improvement in total Q-LES-Q scores (*p* < 0.001) and across multiple domains by week 6 and remained elevated at follow-up. Changes in Q-LES-Q were no longer significant after adjustment for depressive symptoms.

**Conclusion:**

A 6-week course of supervised home-based tDCS was associated with significant QoL improvements in bipolar depression, which appeared to be closely linked to reduction in depressive symptoms. Randomized, sham‐controlled trials are warranted to clarify the specific contribution of tDCS to improve QoL in bipolar depression.

**Supplementary Information:**

The online version contains supplementary material available at 10.1007/s11136-025-04135-2.

## Introduction

Bipolar disorder is a mood disorder characterized by recurrent episodes of (hypo-) mania and depression. Episodes of depression are more frequent and longer lasting than (hypo-) manic episodes [1]. Bipolar depression is often associated with an increased risk of suicidal behaviour [2], profound fatigue, reduced concentration, and diminished motivation, leading to disability and functional impairment across multiple domains, including work or academic responsibilities, household tasks, and interpersonal relationships [3, 4]. Depressive symptoms often have a greater impact on functional impairment than hypo(manic) symptoms [5]. The severity of depressive symptoms is strongly associated with both functional impairment and a poor quality of life (QoL) [6, 7]. However, symptom remission does not necessarily restore QoL [8] and even in euthymic states, patients with bipolar disorder showed reduced QoL compared to healthy controls [9, 10]. Depressive symptoms have been identified as key predictors of QoL in bipolar disorder, and effective management of these symptoms may contribute to significant improvements in patients' well-being [11].

While traditional clinical assessments often focus on symptom severity or psychosocial disability in areas like work or family roles, QoL offers a broader, patient-centered perspective. QoL is now seen as a crucial treatment outcome in bipolar disorder, often holding more personal significance for patients than symptom relief [12]. It captures an individual’s subjective experience of satisfaction and enjoyment across various life areas, including physical, emotional, social, occupational, and even spiritual well-being that is not simply a reflection of their symptoms or medical condition [12, 13]. Importantly, QoL assessments allow patients to identify and prioritize life domains that matter most to them, rather than focusing solely on symptom control [14].

Pharmacological treatments have demonstrated varying degrees of effectiveness in improving QoL among individuals with bipolar disorder. Mood stabilizers like lithium can improve QoL through symptoms stabilization during remission [15] and when paired with psychoeducation [16], but offer no additional benefit when added to optimized personalized treatment [17] and may be less effective than combination therapy [18]. Lamotrigine, especially as adjunctive therapy, improved QoL over 12 weeks [19], while divalproex showed no added benefit [20]. Among atypical antipsychotics, several agents such as quetiapine, olanzapine, lurasidone, and aripiprazole have demonstrated significant improvements in QoL in bipolar depression [18, 21–25], whereas others, including risperidone and ziprasidone, showed only short-term or modest effects [26, 27]. The olanzapine/fluoxetine combination also led to QoL improvements in paediatric bipolar depression, though scores remained lower than in healthy controls [28]. However, these medications raise treatment challenges due to their association with side effects (e.g., antipsychotic-induced weight gain or sedation), high rates of nonadherence, and the risk of manic switching with antidepressant use [29, 30].

In addition to medications, psychosocial interventions can enhance QoL in bipolar disorder. Psychoeducation improves general health and social functioning [16], while Cognitive behavioural therapy (CBT) has demonstrated benefits in vitality and emotional role functioning [14]. Family-focused therapy in adolescents improves physical health and relationships [31] and qualitative findings emphasize the value of routine, identity, and support [32]. Physical activity, at least 150 min per week, has also been linked to significantly higher QoL [33]. Digital tools, such as smartphone-based interventions, show promise for improving biological rhythm regulation and well-being, especially in youth [34, 35]. These therapies are most effective when used alongside medication, though many individuals with bipolar disorder continue to experience residual symptoms or incomplete recovery despite treatment [36, 37]

In recent years, non-invasive brain stimulation (NIBS) techniques, particularly repetitive transcranial magnetic stimulation (rTMS) and transcranial direct current stimulation (tDCS), have been increasingly explored for their potential to improve clinical outcomes, functioning, and QoL in individuals with mood disorders [38]. Studies in major depressive disorder (MDD) have demonstrated significant improvements in QoL following rTMS, as measured by EuroQol 5 Dimensions and Quality of Life Enjoyment and Satisfaction Questionnaire-Short Form (Q-LES-Q-SF) [39–41]. A tele-supervised study of nine sessions of home-based tDCS in MDD increased QoL assessed by Q-LES-Q-SF by 33% from baseline to the follow-up assessment [42]. In our fully remote, double-blind, placebo-controlled randomized controlled trial of 10-week home-based tDCS in MDD, QoL, as measured by the European Quality of Life 5 Dimensions Questionnaire 3 Level version, significantly improved over time in both active and sham groups, although no significant between-group differences were observed [43].

In bipolar disorder, 20 sessions of rTMS have been associated with significant improvements in QoL, as measured by the General Quality of Life Inventory-74 [44] and Global Assessment of Functioning [45, 46], particularly after 2 weeks of treatment [46] and at week 4 [44, 45] compared to sham group. A sham-controlled trial of cranial electrotherapy stimulation in bipolar depression reported a significant post-treatment increase in QoL in the active group [47]. Similarly, 15 sessions of continuous theta-burst stimulation resulted in within-group improvements in QoL, particularly in physical and psychological domains, although differences between the active and sham groups were not statistically significant [48].

tDCS is a novel non-invasive brain stimulation technique that delivers a low-intensity electrical current (typically 1–2 mA) through scalp electrodes, modulating cortical excitability and exerting potential antidepressant effects [49, 50]. The most widely used montage in depression places the anode over the left dorsolateral prefrontal cortex (DLPFC) (F3 international 10–20 EEG system) and the cathode over the right supraorbital or right DLPFC (F4/F8), aiming to upregulate hypoactive prefrontal regions and rebalance dysregulated fronto-limbic networks [50, 51]. In bipolar depression, tDCS has emerged as a promising therapeutic modality, particularly given the limited efficacy and tolerability of existing pharmacological treatments [52]. Meta-analysis of individual patient data suggests that the effects of tDCS treatment peak after approximately 6 weeks and persist for up to 10 weeks beyond sham treatment, supporting the need for longer treatment courses and possibly maintenance sessions to prevent relapse, particularly in bipolar depression [50].

In terms of QoL outcomes, most studies have assessed the QoL as a secondary outcome and found it closely related to depressive symptom change. Significant improvements in the physical and psychological domains of the World Health Organization Quality of Life have been reported following 10 sessions of bifrontal tDCS, delivered with anode over F3 and cathode over F4 at 2 mA for 20 min, in euthymic bipolar disorder compared to sham [53]. In contrast, a large randomized controlled trial using 20 session of 2.5 mA stimulation for 30 min, with anode over F3 and cathode over F8 found no added benefit of tDCS over sham, although QoL measured by Q-LES-Q-SF improved significantly over time in both groups [54]. Similarly, a six-week trial of home-based tDCS in bipolar depression with F3/F4 placement at 2 mA for 30 min reported no significant changes in either mood or QoL, despite high tolerability [55].

As tDCS typically requires repeated sessions over several weeks, in-clinic protocols can raise logistical and accessibility challenges [56]. However, because the technique is well tolerated and has a strong safety profile [52], it can be safely adapted for home use, enabling patients to receive consistent treatment without the burden of frequent clinic visits, while remaining user-friendly, safe, and cost-effective [57]. In the current study, we examined the effect of home-based tDCS on QoL in individuals with bipolar depression. The clinical outcomes of this trial showed significant improvement in depressive symptoms [58]. The present analysis focuses specifically on QoL as a key patient-centered outcome.

## Methods

### Study design and tDCS protocol

The study was an open-label, single-arm acceptability and feasibility trial of home-based tDCS for bipolar depression (ClinicalTrials.gov: NCT05436613 registered on 23 June 2022 https//www.clinicaltrials.gov/study/NCT05436613), approved by the London Fulham Research Ethics Committee (21/LO/0910) and conducted in accordance with the Code of Ethics of the World Medical Association (Declaration of Helsinki). All participants provided electronic informed consent after receiving detailed explanations of the study procedures and having the opportunity to ask questions. Assessments were conducted via videoconference, though participants had the option to attend in person, which none chose to do.

After completing a thorough clinical evaluation, the tDCS equipment (Supplementary Materials) was mailed directly to each participant enrolled in the study. A member of the research team provided real-time guidance on device setup and usage through a video call. The stimulation protocol involved active tDCS sessions lasting 30 min each, using a bifrontal electrode configuration. Specifically, the anodal electrode was positioned over the left DLPFC, and the cathodal electrode was targeting the right DLPFC. Stimulation intensity was set at 2 mA, with a gradual increase over the first 120 s and a ramp-down phase of 15 s at the end. The schedule included five sessions per week over three weeks, followed by two sessions per week for an additional three weeks, resulting in a total of 21 sessions. Participants needed to complete at least 15 sessions to be considered as having completed the intervention. Each session was remotely monitored by a research team member, who kept their camera on to maintain a passive presence. Participants had both their camera and microphone activated to enable communication if support was needed, but interaction was kept minimal unless assistance was requested. During stimulation, participants could engage in quiet activities such as reading or using electronic devices, or they could simply rest.

### Study population

Participants were recruited through online advertisements (77.3%), general practitioner (GP) clinics in primary care (15.9%), and secondary care community mental health teams (6.8%). The healthy control group were recruited through online ads (57.1%) or local outreach (42.9%). Inclusion and exclusion criteria for both groups are provided in Supplementary Materials.

### Clinical assessments

Clinical measures and assessment protocols were described comprehensively in our prior publication [58]. In brief, bipolar depression participants underwent assessments at baseline, week 2, week 6 and at the 18-week follow-up. The healthy control group completed assessments at baseline only. Clinician-rated measures included Montgomery-Åsberg Depression Rating Scale (MADRS) [59] and 17-item Hamilton Depression Rating Scale (HDRS-17) [60] for depression severity, Hamilton Anxiety Rating Scale (HAMA) [61] for anxiety severity, Young Mania Rating Scale (YMRS) [62] for manic symptoms, self-report measure of disability and impairment: Sheehan Disability Ccale (SDS) [63] and self-report measures Patient Health Questionnaire-9 (PHQ-9) [64] for depressive symptoms. Safety was systematically monitored at each visit for any adverse events and with tDCS Adverse Events Questionnaire [65] before and after every session.

### Quality of life assessment

Quality of life enjoyment and satisfaction questionnaire (Q-LES-Q) [66] is a 93-item self-report measure designed to assess QoL from the participant's perspective across eight broad domains: physical/health activities, feelings, leisure time activities, social relations, and general activities, which were scored for all participants. Three domains of work, household duties, school/coursework were scored only for participants to whom these domains applied. Items were rated using a 5-point Likert scale, reflecting the level of enjoyment and satisfaction during the past week. Q-LES-Q assessments were conducted at baseline, week 2, week 6, with a follow-up assessment at week 18 after the initial tDCS session. The questionnaire was shared on the research team's screen, and participants were asked to read each item independently and report their chosen response. The selected number was then highlighted by the researcher. If assistance was requested, the questions were read aloud, this occurred for only one participant.

These raw item scores were summed within each domain and also as an overall total score to generate summary scores. These were then converted into maximum possible score percentage (MPS%) ranging from 0 to 100 to account for differences in the number of items and the number of items completed across domains. Score closer to 0 indicates very poor QoL, while those closer to 100 reflect excellent QoL.

### Statistical analysis

The Q-LES-Q scores were calculated as the percentage maximum possible score achieved by each participant across four time points: baseline (t0), week 2 (t1), week 6, end of treatment period (t2), and week 18, follow-up session (t3). A linear mixed model (LMM) was used to examine changes in Q-LES-Q MPS% scores over time. Time point was modelled as a categorical variable with four levels (baseline, week 2, week 6, and week 18) to account for unequal spacing between assessments. Time point was included as a fixed effect and participant was specified as a random effect to account for within-subject variability. An unstructured covariance matrix was used to model correlations between repeated measures. Fixed effects were assessed using Type III F-tests, and pairwise comparisons were performed on estimated marginal means. Analyses were conducted first without covariates and then repeated with percentage change in MADRS scores included as a covariate to control for changes in depressive symptoms.

Comparisons of demographic variables between the bipolar depression group and the healthy control group were conducted using independent samples t-tests for continuous variables (age, years of education, and IQ), and a Chi-square test for the categorical variable (gender). To evaluate differences in Q-LES-Q scores between participants with bipolar depression and healthy controls, independent samples t-tests were performed. All statistical procedures were carried out using IBM SPSS Statistics for Mac, version 29.0. Analyses were two-tailed, with statistical significance set at *p* = 0.05.

## Results

### Participants

A total of 44 participants with bipolar depression (31 women) were enrolled, with a mean age of 47.27 ± 12.94 (mean ± standard deviation) years and a mean duration of illness of 18.98 ± 12.47 years. 41 participants (93.2%) (mean age 47.93 ± 13.15 years) completed the full 6-week course of treatment. Baseline demographic characteristics, clinical data and treatment during trial are detailed in Supplementary Materials.

Healthy controls participants consisted of 28 adults (17 women) with a mean age of 44.68 ± 14.45 years. The bipolar depression and healthy control groups were comparable in demographic characteristics, with similar distributions in age (47.27 ± 12.9 vs. 44.68 ± 14.45 years), education (16.30 ± 2.46 vs. 16.89 ± 2.11 years), and IQ (100.66 ± 9.3 vs. 103.39 ± 8.77).

### Clinical outcomes

Clinical outcomes are available in full details in our prior publication [58]. In summary, depressive symptoms, as measured by MADRS, showed marked reduction by week 6 (M = 8.91, SD = 5.56), with 77.3% of participants meeting criteria for clinical response and 47.7% achieving remission. Significant improvements from baseline were also observed in HDRS-17, HAMA, YMRS, PHQ-9, and SDS scores. The most common adverse effects were tingling (83.5%), skin redness (40.6%), itching (29.3%), and a burning sensation (26.5%). A total of 90.6% of adverse events related to tDCS were rated as mild, 9% as moderate, and 0.4% as severe [58].

### Quality of life

**Fig. 1:**
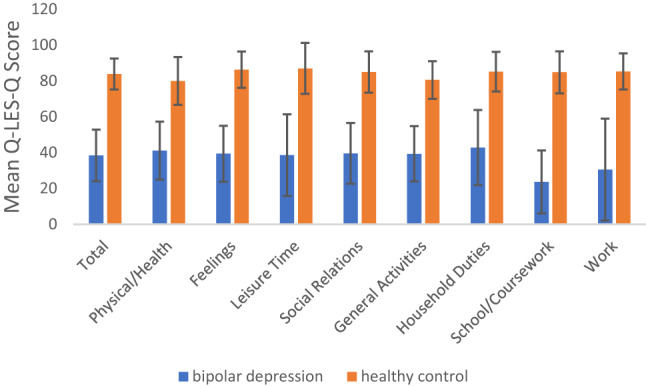
Comparison of Q-LES-Q domain scores between participants with bipolar depression and healthy controls at baseline. Mean scores across all domains were significantly lower in the bipolar group compared to healthy controls, *p* < 0.001

At baseline, participants with bipolar depression reported significantly lower QoL across all Q-LES-Q domains compared to healthy controls (all *p* < 0.001; Fig. 1), with total Q-LES-Q scores substantially lower in the bipolar group (M = 38.46, SD = 14.42) compared to controls (M = 83.86, SD = 8.64; t(66.05) = -16.32, *p* < 0.001; Table 1).

**Table 1:** Comparison of baseline Q-LES-Q scores between participants with bipolar depression and healthy controls

	BD (n)	BD Mean (SD)	HC (n)	HC Mean (SD)	t	*P*-value	d
total score	41	38.46 ± 14.42	28	83.86 ± 8.64	− 10.52	< 0.001***	− 3.49
physical/health activities	41	41.15 ± 16.15	28	80.00 ± 13.31	− 15.14	< 0.001***	− 2.58
feelings	41	39.41 ± 15.60	28	86.29 ± 10.09	− 15.14	< 0.001***	− 3.43
leisure activities	40	39.60 ± 22.20	28	87.00 ± 14.20	− 10.85	< 0.001***	− 2.45
social relations	41	39.59 ± 16.95	28	84.96 ± 11.52	− 12.34	< 0.001***	− 3.02
general activities	41	47.98 ± 17.20	28	80.57 ± 10.49	− 12.33	< 0.001***	− 2.19
work	21	51.43 ± 16.59	20	87.55 ± 11.90	− 9.78	< 0.001***	− 2.49
household duties	37	44.16 ± 20.29	24	85.33 ± 11.19	− 10.45	< 0.001***	− 2.38
school/coursework	9	34.22 ± 7.46	6	84.83 ± 11.72	− 7.72	< 0.001***	− 5.43

Mean values are presented with ‘±’ standard deviation. *P*-value represent two-sided t-test. BD, bipolar depression; HC, healthy control. *p < 0.05, **p < 0.01, ***p < 0.001

**Fig. 2:**
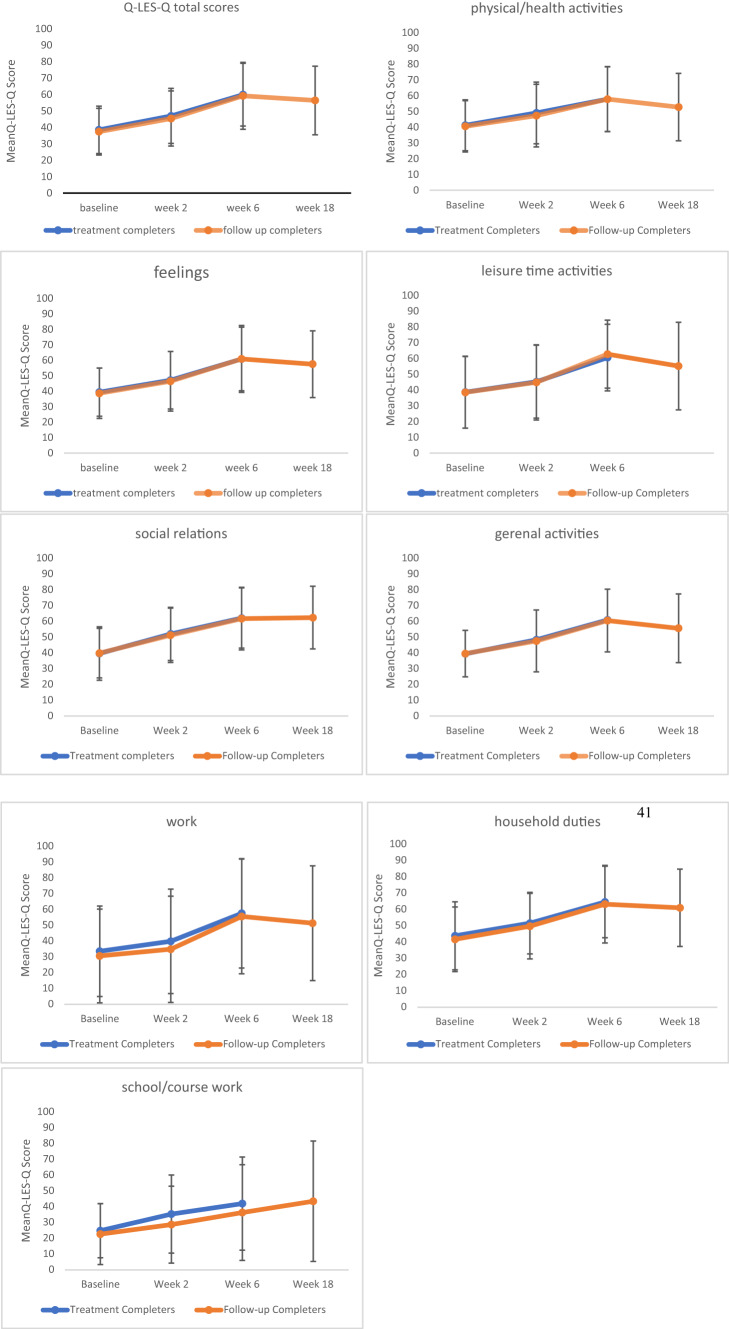
Change in Q-LES-Q scores across four time points. Error bars represent standard deviations. Q-LES-Q percentage maximum scores range from 0 to 100, with higher values indicating greater QoL

In the bipolar depression group, the results showed a significant effect of time point on Q-LES-Q total scores, F_(3, 37.26)_ = 21.99, *p* < 0.001, indicating that total scores changed significantly over the course of the treatment (Fig. 2). Estimated marginal means showed that total scores increased from 38.46 (SE = 2.25) at baseline to 46.95 (SE = 2.61) at week 2, and peaking at end of treatment 59.85 (SE = 2.98), and remaining relatively stable at follow-up 56.96 (SE = 3.32). Pairwise comparisons revealed that all changes from baseline to later time points were statistically significant (all *p* < 0.001), except between final treatment session and the follow-up (*p* = 1.000). After controlling for depressive symptom severity (percentage changes in MADRS scores), the effect was no longer statistically significant, F_(3, 35.58)_ = 1.740, *p* = 0.176, suggesting that improvements in QoL were at least partially attributable to reductions in depressive symptoms.

Significant improvements were observed across most Q-LES-Q domains from baseline to follow-up (Table 2). Physical/health activities domain showed a substantial increase in scores, rising from 41.14 (SE = 2.52) to 48.58 to 57.73 (SE = 3.22) at the end of treatment. Feelings domain also improved from 39.41 (SE = 2.43) at baseline to 60.85 (SE = 3.21) at week 6, (F_(3,40)_ = 20.17, *p* < 0.001). A similar trend was found in leisure time activities, which rose from 38.63 (SE = 3.5) to 60.53 (SE = 3.30) by end of treatment (F_(3,40)_ = 12.13, *p* < 0.001). Social relations also improved significantly over time (F_(3,40)_ = 15.31, *p* < 0.001), increasing from 39.58 (SE = 2.64) to 62.63 (SE = 3.27). Likewise, the general activities domain increased from 39.39 (SE = 2.40) at baseline to 60.87 (SE = 2.93) at week 6 (F_(3.40)_ = 18.66, *p* < 0.001).

**Table 2:** Mean change for Q-LES-Q percentage maximum scores from baseline to follow-up session

Q-LES-Q domain	Mean change	F-value	*p*-value	Significant after controlling for MADRS
total score	38.46 ± 2.25 → 46.95 ± 2.61 → 59.85 ± 2.98 → 56.96 ± 3.32	21.99	< 0.001***	No
physical/health activities	41.14 ± 2.52 → 48.58 ± 3.05 → 57.73 ± 3.22 → 55.00 ± 3.19	17.44	< 0.001***	No
feelings	39.41 ± 2.43 → 47.09 ± 2.90 → 60.85 ± 3.21 → 57.95 ± 3.12	20.17	< 0.001***	No
leisure time activities	38.63 ± 3.55 → 45.26 ± 3.61 → 60.53 ± 3.30 → 59.92 ± 3.91	12.13	< 0.001***	No
social relations	39.58 ± 2.64 → 51.97 ± 2.62 → 62.07 ± 2.97 → 62.63 ± 3.27	15.31	< 0.001***	No
general activities	39.39 ± 2.40 → 48.29 ± 3.11 → 60.87 ± 2.93 → 56.36 ± 3.11	18.66	< 0.001***	No
work	31.97 ± 4.62 → 43.04 ± 5.31 → 54.24 ± 5.31 → 50.45 ± 5.29	10.92	< 0.001***	No
household duties	43.70 ± 3.29 → 51.14 ± 3.04 → 62.48 ± 3.40 → 64.78 ± 3.41	12.68	< 0.001***	No
school/coursework	31.47 ± 4.03 → 34.83 ± 4.80 → 36.93 ± 3.97 → 35.03 ± 5.34	0.45	0.720	No

Mean values are presented with ‘±’ standard error, *p < 0.05, **p < 0.01, ***p < 0.001

Three domains of work, household duties, school/coursework were scored only for participants to whom these domains applied. The work domain showed significant improvement over time (F_(3,39)_ = 10.92, *p* < 0.001), increasing from 31.97 (SE = 4.62) at baseline to 54.24 (SE = 5.31) at week 6. Household duties showed steady improvement across all time points, from 43.70 (SE = 3.29) to 62.48 (SE = 3.40) at end of treatment (F_(3,40)_ = 12.68, *p* < 0.001). School/coursework domain did not change significantly (F_(3,12)_ = 0.45, *p* = 0.720), with means of 31.47 (SE = 4.03) to 36.93 (SE = 3.97) by end of treatment. After controlling for percentage changes in MADRS scores, the effect of time was no longer significant for any of the Q-LES-Q domains.

**Fig. 3:**
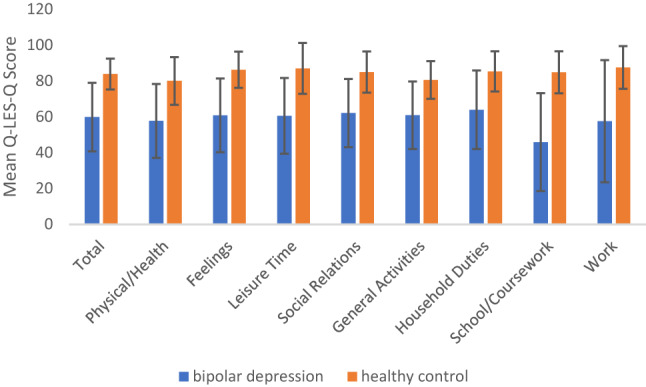
Comparison of Q-LES-Q domain scores between participants with bipolar depression and healthy controls at end of treatment. Mean scores across all domains were significantly lower in the bipolar group compared to healthy controls, *p* < 0.001

At the end of treatment, Q-LES-Q scores in participants with bipolar depression remained significantly lower than healthy controls across all domains (Fig. 3). Post-treatment total Q-LES-Q score was significantly lower in the bipolar group (M = 59.85, SD = 19.09) than controls (M = 83.86, SD = 8.64, t(59.63) = -7.06,* p* < 0.001; Table 3). However, after controlling for change in MADRS score, the group difference in Q-LES-Q total score became non-significant (F_(1.65)_ = 0.88, *p* = 0.351).

**Table 3:** Comparison of post-treatment Q-LES-Q scores between participants with bipolar depression and healthy controls

	BD (n)	BD Mean (SD)	HC (n)	HC Mean (SD)	t	*P*-value	d
total score	41	59.85 ± 19.09	28	83.86 ± 8.64	− 7.06	< 0.001***	− 1.53
physical/health activities	41	57.73 ± 20.62	28	80.00 ± 13.31	− 5.45	< 0.001***	− 1.24
feelings	41	60.85 ± 20.56	28	86.29 ± 10.10	− 6.81	< 0.001***	− 1.48
leisure activities	41	60.54 ± 21.13	28	87.00 ± 14.21	− 5.79	< 0.001***	− 1.42
social relations	41	62.07 ± 19.05	28	84.96 ± 11.52	− 6.21	< 0.001***	− 1.39
general activities	41	60.88 ± 18.81	28	80.57 ± 10.49	− 5.56	< 0.001***	− 1.23
work	32	57.56 ± 34.08	20	87.55 ± 11.91	− 4.55	< 0.001***	− 1.06
household duties	38	63.92 ± 21.91	24	85.33 ± 11.19	− 5.07	< 0.001***	− 1.14
school/coursework	10	45.90 ± 27.32	6	84.83 ± 11.72	− 3.55	0.063	− 0.85

Mean values are presented with ‘±’ standard deviation. *P*-value represent two-sided t-test. BD, bipolar depression; HC, healthy control. * *p* < 0.05, ** *p* < 0.01, *** *p* < 0.001

## Discussion

Individuals with bipolar disorder consistently report reduced QoL across multiple domains, including social relationships, work productivity, and emotional well-being, even during euthymic phases [9] and in the early stage of illness [67]. Depression severity is shown to be strongly associated with lower QoL [7]. In contrast to symptom-based assessments, QoL measures can also reflect aspects of daily life from the patients’ perspective that may be overlooked in clinical evaluations [14].

In this study, we examined the impact of home-based tDCS on QoL among individuals with bipolar depression, using both total and domain-level Q-LES-Q scores. A 6-week course of home-based tDCS with real-time supervision was associated with significant improvements across multiple domains of QoL by the end of treatment. Notably, several of these improvements were maintained at the follow-up, indicating sustained benefits. The effect of time on QoL were no longer statistically significant after controlling for change in depressive symptoms, suggesting that improvements in QoL are closely linked to reductions in depressive symptoms [68, 69]. As depressive symptoms in bipolar disorder are associated with hypoactivity of left DLPFC and dysregulated fronto-limbic connectivity, anodal tDCS over left DLPFC aims to upregulate prefrontal excitability and restore balance in these networks [70]. This neuromodulation strengthens prefrontal networks, improving their regulation of emotion- related brain regions such as anterior cingulate cortex and amygdala, and helping to reduce negative affect [50]. By improving cognitive control and emotion regulation, tDCS may not only reduce depressive symptoms but also promote functional recovery and better daily life participation, thereby contributing to improvements in QoL [5]. This pattern is consistent with a wide range of literature across different intervention types including pharmacological treatment [19, 23, 24], psychoeducational interventions [14, 16], and in NIBS such as TMS [44–46] and tDCS [53]. These studies highlight the role of depressive symptom severity in QoL outcomes, and report strong associations between improvements in mood and enhanced perceived QoL [6, 69, 71].

Improvement in symptoms does not always translate into proportional improvements in QoL [8]. This has been observed in pharmacological studies, where mood stabilizers or antipsychotics improved symptoms without corresponding QoL gains [17, 20]. Our RCT of home-based tDCS in MDD showed significant improvement in depressive symptoms compared to sham stimulation but there were no significant differences between groups on QoL scale [43].

When we compared QoL in bipolar depression with the healthy control group, consistent with previous literature [14, 71, 72], our results indicated that individuals with bipolar disorder had significantly lower QoL compared to healthy controls at baseline and after end of treatment, despite significant clinical improvement in depressive symptoms within the bipolar depression group.

While our findings suggest that improvements in QoL were largely influenced by reductions in depressive symptoms, previous research indicates that symptom relief alone may not be sufficient for full functional recovery and are not limited to symptomatic phases. A meta-analysis comparing QoL in euthymic bipolar patients with healthy controls mentioned that QoL in euthymic bipolar disorder patients remains impaired even in the absence of mood symptoms, suggesting a trait-like component [73]. In the current study, although depressive symptom reduction statistically accounted for improvement in QoL, the bipolar depression group continued to report lower QoL than controls at the end of the treatment, suggesting residual deficits that may not be solely attributable to mood state. This was observed despite significant clinical gains following tDCS intervention. These findings highlight that while symptom relief is important, it may not be sufficient to fully restore overall functioning.

Limitations of the study include the absence of a sham control group, which restricts the ability to distinguish the specific effects of active tDCS from sham condition. The relatively small sample size, combined with a predominantly white and female participant pool, limits the generalizability of the findings to more diverse populations. Another potential limitation is the influence of therapeutic contact, which may have contributed to the high response and remission rates. Our thematic analyses of participants’ views on home-based tDCS from the current study [74] and from our previous study in unipolar depression [75] have suggested that the consistent presence of the same researcher at each visit, built a sense of safety and connection, which may have enhanced treatment engagement and symptom improvement [76]. Furthermore, the study did not control for concurrent psychotherapy or the types of medications participants were taking. Although participants were required to either maintain a stable dosage of mood-stabilizing medication for at least two weeks or abstain entirely, the lack of control over pharmacotherapy introduces potential confounds.

## Conclusion

In conclusion, home-based tDCS with real-time remote supervision was associated with improvement in QoL in bipolar depression, with benefits sustained beyond the treatment period. Our findings contribute to the growing body of evidence showing that improvements in QoL are closely associated with reductions in depressive symptoms, while also raising the possibility that tDCS may contribute to QoL through mechanisms not entirely explained by mood improvement. These findings suggest potential benefits of tDCS for improvement of QoL in bipolar depression and should be further investigated in a randomized, sham‐controlled design.

## Supplementary Information

Below is the link to the electronic supplementary material.

Supplementary Material

## Data Availability

The anonymised datasets used and/or analysed during the current study are available from the corresponding author on reasonable request.

## Abbreviations

BD: Bipolar Depression
CBT: Cognitive Behavioural Therapy
HC: Healthy Control
HAMA: Hamilton Anxiety Rating Scale
HDRS-17: 17-Item hamilton depression rating scale
mA: milliampere
MADRS: Montgomery-Åsberg Depression Rating Scale
MDD: Major Depressive Disorder
MINI: Mini-International Neuropsychiatric Interview
MPS%: Maximum Possible Score percentage
NIBS: Non-Invasive Brain Stimulation
rTMS: Repetitive Transcranial Magnetic Stimulation
SDS: Sheehan Disability Scale
tDCS: transcranial Direct Current Stimulation
PHQ-9: Patient Health Questionnaire-9
QoL: Quality of Life
Q-LES-Q: Quality of life enjoyment and satisfaction questionnaire
Q-LES-Q-SF: Quality of life enjoyment and satisfaction questionnaire-short form
YMRS: Young mania rating scale
