# Supplementary material for: Home-based transcranial direct current stimulation (tDCS) for bipolar depression: effects on quality of life and functioning—an open-label study
Source: Qual Life Res. 2026 Jan 9;35(2):33. doi: 10.1007/s11136-025-04135-2 (PMC12789176; doi:10.1007/s11136-025-04135-2)
Supplement: Supplementary file 1 — Supplementary Material [file 11136_2025_4135_MOESM1_ESM.docx]

**Supplementary Materials**

**Title:**

Home-Based Transcranial Direct Current Stimulation (tDCS) for Bipolar Depression: Effects on Quality of Life and Functioning: an open-label study

**Authors:**

Hakimeh Rezaei ^a, b, c^, Rachel D. Woodham ^b, c^, Ali-Reza Ghazi-Noori ^c^, Elvira Bramon ^d^, Michael Bauer ^a^, Allan H. Young ^b,^ ^e, f^, Cynthia H.Y. Fu* ^b, c, e^, Philipp Ritter* ^a, b^

**Affiliation:**

1. Department of Psychiatry and Psychotherapy, Faculty of Medicine, Technische Universität Dresden, Dresden, Germany
2. Institute of Psychiatry, Psychology and Neuroscience, King’s College London, London, UK
3. School of Psychology, University of East London, London, UK
4. Department of Psychiatry, University College London, London, UK
5. National Institute for Health Research Biomedical Research Centre at South London and Maudsley NHS Foundation Trust, King's College London, London, UK
6. South London and Maudsley NHS Foundation Trust, Bethlem Royal Hospital, Beckenham, UK

*shared last-authorship

Table of Contents

[Figure 1. tDCS headset and electrode positioning diagram 3](#_Toc210027449)

[Inclusion and exclusion criteria 4](#_Toc210027450)

[Table 1 Baseline demographic and clinical data 6](#_Toc210027451)

[References 7](#_Toc210027452)

**Figure 1.** tDCS headset and electrode positioning diagram.

(A) Figure depicting tDCS headset (Flow Neuroscience, Sweden). (B) The targeted stimulation locations at F3 and F4 are shown, with the anode in blue and the cathode in red, according to the International 10-20 EEG System, using 22.9 cm² electrodes.

# **Inclusion and exclusion criteria**

Inclusion criteria-bipolar disorder group:

1. adults aged 18 or older
2. diagnosis of bipolar disorder, defined by Diagnostic Statistic Manual of Mental Disorders, Fifth Edition (DSM-5)[1], in a structured clinical assessment using the Mini-International Neuropsychiatric Interview (MINI; Version 7.0.2) [2]
3. moderate or greater depressive symptoms as measured by a minimum score of 18 on Montgomery-Åsberg Depression Rating Scale (MADRS) [3]
4. on a stable dosage of mood-stabilizing medication for a minimum of two weeks or medication free for a minimum of two weeks.

Exclusion criteria:

1. any concurrent psychiatric disorder, including obsessive compulsive disorder
2. substantial suicide risk, assessed by Suicidality module of the Mini-International Neuropsychiatric Interview [2], Montgomery-Åsberg Depression Rating Scale and 17-item Hamilton Depression Rating Scale (HDRS-17) [4]
3. presence of manic/hypomanic symptoms as measured by score greater than 8 on Young Mania Rating Scale (YMRS) [5]
4. having a scalp or skin conditions, metallic implants
5. history of epilepsy
6. history of seizures with loss of consciousness
7. history of neurological disorder or history of migraines

Healthy control group:

- adults aged 18 or older
- excluded if:
  - personal or familial psychiatric history
  - significant suicide risk
  - manic or hypomanic symptoms (YMRS > 8)

# **Table 1** Baseline demographic and clinical data

|  | BD group  Mean ± SD | HC group  Mean ± SD |
| --- | --- | --- |
| Total number (Female) | 44 (31) | 28 (17) |
| Mean Age (years) | 47.27 ± 12.9 | 44.68 ± 14.45 |
| Age range (years) | 24-76 | 21-72 |
| Years of education | 16.30 ± 2.46 | 16.89 ± 2.11 |
| IQ | 100.66 ± 9.3 | 103.39 ± 8.77 |
| Clinical rating | | |
| MADRS | 24.59 ± 2.64 | 0.75 ± 1.07 |
| HDRS-17 | 19.98 ± 2.62 | 0.82 ± 1.44 |
| HAMA | 16.55 ± 5.26 | 0.25 ± 0.51 |
| PHQ-9 | 16.80 ± 4.94 | 1.36 ± 1.54 |
| SDS | 20.77 ± 5.87 | 0.46 ± 0.92 |
| Duration of illness (years) | 18.98 ± 12.47 |  |
| Duration current depressive episode (weeks) (range) | 49.55 ± 100.4 |  |
| Previous number of episodes | 18.16 ± 16.13 |  |
| Treatments during trial | |  |
| Taking mood stabilizer and other medications (Lamotrigine, Lithium, Quetiapine, Olanzapine, Aripiprazole) | 38 (86%) |  |
| Taking antidepressant medication only | 1 (2.3%) |  |
| Taking no medication | 5 (11.4%) |  |
| Engaged in psychotherapy (either CBT or psychodynamic psychotherapy) | 12 (27.3%) |  |

Categorial variables are presented as number of participants with percentage in parentheses for treatment during trial. Mean values are presented with ± standard deviation; BD, bipolar depression; HC, healthy control; Montgomery-Åsberg Depression Rating Scale; HDRS-17, Hamilton Depression Rating Scale; HAMA, Hamilton Anxiety Rating Scale [6]; YMRS, Young Mania Rating Scale; PHQ-9, Patient Health Questionnaire-9 [7, 8]; SDS, Sheehan Disability Scale [8]

# **References**

1. American Psychiatric Association. (2013). *Diagnostic and Statistical Manual of Mental Disorders*. American Psychiatric Association. https://doi.org/10.1176/appi.books.9780890425596

2. Sheehan, D. V, Lecrubier, Y., Sheehan, K. H., Amorim, P., Janavs, J., Weiller, E., Hergueta, T., Baker, R., Dunbar, G. C. (1998). The Mini-International Neuropsychiatric Interview (M.I.N.I.): the development and validation of a structured diagnostic psychiatric interview for DSM-IV and ICD-10. *The Journal of clinical psychiatry*, *59 Suppl 20*, 22-33;quiz 34-57.

3. Montgomery, S. A., & Åsberg, M. (1979). A New Depression Scale Designed to be Sensitive to Change. *British Journal of Psychiatry*, *134*(4), 382–389. https://doi.org/10.1192/bjp.134.4.382

4. Hamilton, M. (1960). A RATING SCALE FOR DEPRESSION. *Journal of Neurology, Neurosurgery & Psychiatry*, *23*(1), 56–62. https://doi.org/10.1136/jnnp.23.1.56

5. Young, R. C., Biggs, J. T., Ziegler, V. E., & Meyer, D. A. (1978). A Rating Scale for Mania: Reliability, Validity and Sensitivity. *British Journal of Psychiatry*, *133*(5), 429–435. https://doi.org/10.1192/bjp.133.5.429

6. Hamilton, M. (1959). The Assessment of Anxiety States by Rating. *British Journal of Medical Psychology*, *32*(1), 50–55. https://doi.org/10.1111/j.2044-8341.1959.tb00467.x

7. Kroenke, K., Spitzer, R. L., & Williams, J. B. W. (2001). The PHQ-9. *Journal of General Internal Medicine*, *16*(9), 606–613. https://doi.org/10.1046/j.1525-1497.2001.016009606.x

8. Sheehan DV. (1983). The Sheehan disability scales. Anxiety dis. Overcome it. *New York: Charles Scribner and Sons*, 151.
